# The factor structure of the Center for Epidemiological Study - Depression Scale in people with multiple sclerosis

**DOI:** 10.12688/f1000research.25129.1

**Published:** 2020-08-25

**Authors:** Ian I. Kneebone, Chris Fife-Schaw, Lawrence T. Lam, Roshan das Nair

**Affiliations:** 1 Discipline of Clinical Psychology, Graduate School of Health, University of Technology Sydney, Ultimo, NSW, 2007, Australia; 2 School of Psychology, University of Surrey, Guildford, Surrey, GU2 7XH, UK; 3 Vice President (Academic), Tung Wah College, Hong Kong, Homantin, SAR, Hong Kong; 4 Division of Psychiatry and Applied Psychology, School of Medicine, University of Nottingham, Lenton, Nottingham, Nottinghamshire, NG7 2RD, UK

**Keywords:** Multiple Sclerosis, Depression, Center for Epidemiological Study - Depression Scale, Factor Analysis

## Abstract

**Background:** Depression is common in multiple sclerosis (MS); however, its assessment is complicated by biological processes. In this context it is important to consider the performance of depression screening measures including that their factor structure is consistent with expectation.  This study sought to identify the factor structure of the Center for Epidemiological Study - Depression Scale (CES-D) in people with MS (PwMS).

**Methods:** Participants (N = 493) were those who had consented to take part in a large three-phase longitudinal study of depression in PwMS. CES-D questionnaires completed at phase 1 of the study were utilised. An error in the questionnaire meant it was most appropriate to consider data for 19 of the 20 CES-D questionnaire items. The data was split into two samples by a random selection process to create an exploratory, model development sample and a validation sample. The first sample was subject to confirmatory factor analysis. Following examination of model fit and specification errors, the original model was modified. The revised model was tested in the confirmation sample to assess reproducibility.

**Results:** The analysis results supported the original four factor solution for the CES-D, that is: Depressed Affect, Positive Affect, Somatic Complaints/Activity Inhibition, and Interpersonal Difficulties.

**Conclusions:** The CES-D appears to have a coherent structure with which to examine depression in PwMS.

## Introduction

Multiple sclerosis (MS), a disease of the central nervous system, involves a variety of debilitating physical, sensory, cognitive and emotional symptoms. People with MS (PwMS) are typically diagnosed aged 20–40 years and often face psychosocial consequences including disruptions to life goals, education/employment, income, relationships, leisure activities, and daily living activities ^1^. Indeed, MS is considered the leading cause of disability in young adults in the developed world ^2^. Furthermore, the chronic and unpredictable course of MS and side effects of MS-related medications have profound social and psychological consequences ^3^.

Depression is common in PwMS with a point prevalence rate of up to 40% and up to 50% experiencing it at some time post diagnosis ^4, 5^. Depression is also associated with higher suicidal ideation and attempts in pwMS ^6^ and often disrupts relationships and reduces compliance with MS disease-modifying treatments ^7^. The negative sequelae associated with depressive symptoms in MS also include decreased perceived cognitive function ^8^, increased fatigue ^9^, and sleep difficulties ^10^.

Depressive symptoms in MS may not only be caused by the psychosocial adjustment to the illness and its affects but in relate to biological aspects of the disease ^11^. Organic changes, including in, neuroendocrine function, inflammatory process and brain associated brain damage likely to play a role ^12– 14^.

Depression is assessed by clinical interviews but is more often assessed (especially in research contexts) through self-report questionnaires. There is a significant overlap between the somatic symptoms common to depression and MS symptoms, principally fatigue, poor sleep and concentration. This overlap has led to concerns over the accuracy of assessment of depression in PwMS ^15^. Furthermore, some self-report measures of depression include questions about health and work difficulties, which are also impacted by MS-related physical disability ^16^. As such, levels of depression in MS may be over- or underestimated, particularly when using self-report measures.

Despite the Center for Epidemiological Studies - Depression Scale (CES-D) ^17 ^ originally being developed for use with the general community ^18^ it has become to widely used in clinical research and practice settings, including with PwMS ^19^. The original CES-D is a 20-item self-reported scale that has been shown to measure depressive symptoms across four domains: Depressed Affect, Positive Affect, Somatic Complaints/Activity Inhibition, and Interpersonal Difficulties ^18^. The four original latent factors have been replicated in numerous populations ^20^. However, a number of other studies have yielded different CES-D factor structures. For instance a three-factor solution was evident in Chinese adolescents ^21^ and as many as five factors has been found in a random sample of adults in the USA ^22^. Some studies have also shown the presence of variation in the items comprising each factor ^23, 24^ and the magnitude of item loadings has varied across clinical groups ^25^. Variations such as these may affect the sensitivity and specificity of this instrument in detecting depressive symptoms in different populations and question the test’s validity. With respect to PwMS, Amtmann *et al*. ^26^ confirmed acceptable inter-item reliability and convergent/discriminant validity of the CES-D in a sample of 455 patients. However, in this study, confirmatory factor analysis was only used to consider the presence of a single factor, depression, i.e., unidimensionality. One study considered the multi-dimensional factor structure of the French version of the CES-D in people with MS, confirming the initially identified four factor structure ^27^.

Consistent with concerns that different populations can produce different factor analytic structures for measures ^28, 29^ and this pertains to validity ^30^, the current study aimed to assess whether the four-factor model of depression is supported in PwMS in an English language version of the CES-D.

## Methods

The research was approved by the University of Surrey Advisory Committee on Ethics [ACE/99/30/Psych]. Participants were those who had provided written, informed consent to take part in a large three-phase longitudinal study of depression in PwMS commencing in 1999 ^31^ Participants were required to have a diagnosis of MS and be 18 years or older. Only participants under the age of 65 years were included in the current study. Participants were a convenience sample who self-referred to the study following the publication of an article in an MS magazine available to people in the United Kingdom. Data, including the CES-D, were collected yearly by postal survey using a prepaid system, on three occasions. The CES-D data collected at phase 1 are reported here. No power analysis to determine sample size was calculated a priori.

### Measures

The CES-D ^17^ requests the self-reporting of depressive symptoms experienced over the previous week. The 20 items are rated on a ‘0’ to ‘3’ scale, with a higher rating indicating greater symptom frequency. Scores can range from 0 to 60. The CES-D is considered to be relatively unaffected by somatic variables ^32^ and has been used in studies considering depression in PwMS ^19^.

### Data analysis

Data analysis was performed using SPSS v25. The data set was split into two by a random selection process to create an exploratory model development sample and the validation sample. In the exploratory stage, a model based on Radloff’s ^17^ original specification of the CES-D was tested using confirmatory factor analysis (CFA). Model fit and the degree of specification errors were examined and modifications of the original model made as indicated and re-tested in the exploratory sample. This specification search involved examination of modification indices, identification of non-significant paths and conceptual acceptability. In the second stage, the revised model was tested in the confirmation sample to assess reproducibility. Models were estimated using maximum likelihood estimation and adequacy of fit was assessed using criteria proposed by Hu and Bentler ^33^ for CFA models. These criteria of goodness of fit are a non-normed fit index/Tucker Lewis index (NNFI/TLI) >0.95, comparative fit index (CFI) >0.95, root mean square error of approximation (RMSEA) <0.06 and standardized root mean square residual (SRMR) <0.08. For completeness we also report the traditional chi-square fit index and the reduced chi-squared statistics (χ ^2^/df), which should ideally be less than 2 ^34^. To determine internal reliability of each factor Cronbach’s alpha coefficients were calculated. Due to a typographical error in one of the items (item 10: ‘I felt fearful’ was printed as ‘I felt tearful’) in the questionnaire used ^a^, which was only observed after data collection; this item had to be removed, and the analyses are reported on the 19 items. The exploratory and confirmatory factor analyses were performed again on the full 20 items.

## Results

### Participants

A total of 493 participants were recruited (n = 399 women). The age range was 22 years to 65 years (mean, X = 45.8 years, SD = 9.25). MS diagnoses sub-types were as follows: 45% had ‘relapsing remitting type’, 20% had ‘secondary progressive type’, 10% has ‘primary progressive type’, and 19% ‘did not know’ their MS sub-type, with 5% ‘missing data’. Over half (62.9%) of participants scored >16 on the CES-D, the cut off indicating ‘significant depressive symptoms’ (X = 22.1, SD = 12.57, Range 0–59).

### Data set

Since there was only one sample for factor analyses, both exploratory and confirmatory, the original data set consisting of 493 patients was split into two using SPSS’s random selection process. Only cases with complete data on all 20 CES-D items (n = 472) were retained and as a result, a model specification sample with 235 participants was generated and the remaining 237 were used as the validation sample. 

### Confirmatory factor analysis in the exploration sample

Following Radloff’s ^17^ exploratory factor analyses we assessed the fit of our data to her original model with the omission of item 10. Table 1 gives the fit indices for this initial model (Model 1). These indices suggest that the model, while not unreasonable in terms of the absolute size of the indices, is mis-specified. A specification search suggested that the two Somatic Symptoms items ‘I felt everything that I did was an effort (CESD7)’ and ‘I could not get going (CESD20)’ shared variance not entirely captured by the Somatic Symptoms factor (MI = 10.21) so a second model with this term added was assessed. Fit indices for this model (Model 2) are presented in Table 1. On Hu and Bentler’s ^33^ four criteria this model fits acceptably and the χ ^2^/df criterion is also satisfactory. No further modifications were made as these either made trivial improvements in fit or did not make sense theoretically.

**Table 1.** Goodness-of-fit statistics for the models tested – with item 10 removed.

Model	Description	χ ^2^	Df	χ ^2^/df	NNFI/TLI	CFI	RMSEA	SRMR
1	As specified in Radloff (1977, minus Item 10)	233.05	146	1.60	.948	.955	.050	.048
2	Model 1 with correlated error terms for items CESD7 and CESD20	218.60	145	1.51	.955	.962	.047	.046
3	Model 2 tested with the confirmation sample data	213.84	145	1.48	.959	.965	.045	.046

**Figure 1.**
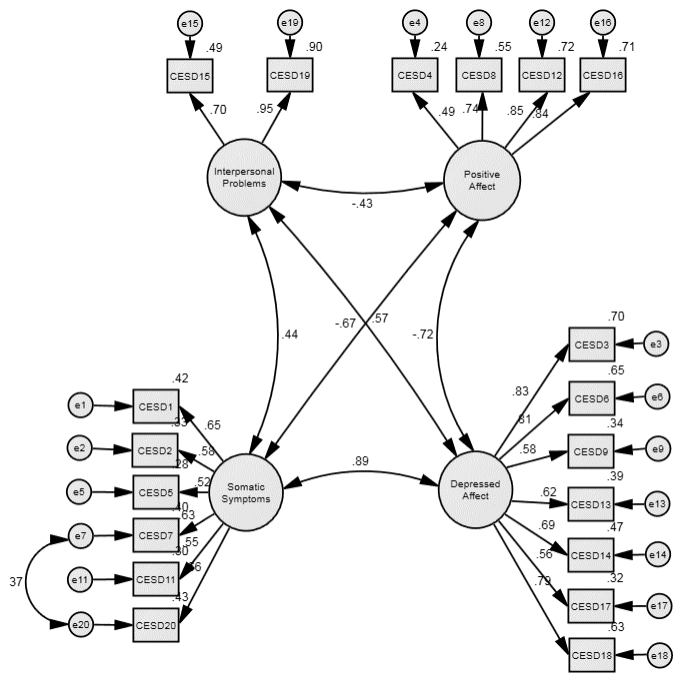
Revised CES-D model for People with MS – with item 10 removed. Figures are standardised maximum likelihood estimates.

The re-specified model was then assessed in the confirmation sample (Model 3) and as seen in Figure 1, the fit indices suggest a satisfactory fit of this modified four-factor model. Indeed, the fit indices are slightly better in the confirmation sample than the exploratory one. The modification made in the re-specification process did not imply factorially complex items nor that the basic four-factor CES-D model was substantively incorrect, so we estimated the standard Cronbach’s Alpha reliability coefficients for the implied CES-D subscales in the confirmation sample. These were 0.81 for Positive Affect, 0.87 for Depressed Affect, 0.73 for Somatic Symptoms and 0.79 for Interpersonal Problems.

**Figure 2.**
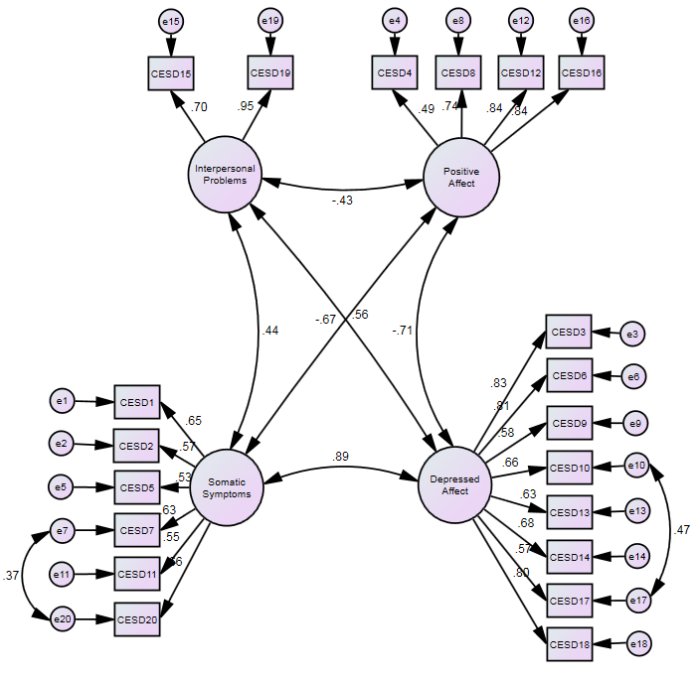
Revised CES-D model for People with MS – with all 20 items included. Figures are standardised maximum likelihood estimates.

For the 20-item scale, following Radloff’s ^17^ exploratory factor analyses we assessed the fit of our data to her original model. Table 2 gives the fit indices for this initial model (Model 1). These indices suggest that the model, while not unreasonable in terms of the absolute size of the indices, is mis-specified. A specification search suggested a substantial correlation between the error terms associated with the depressed affect items ‘I felt tearful (CESD10)’ and ‘I had crying spells (CESD17)’ needed to be modelled (MI = 51.92) so a second model with this term added was assessed. This correlated error term reflects the incorrect wording of Item 10 we identified in the Johnston *et al*. ^35^ version of the CES-D we used. Fit indices for this model (Model 2) are presented in Table 2. On three of Hu and Bentler’s ^33^ four criteria this model fits acceptable in the χ2/df is also satisfactory. The addition of this path in the model suggests that these two items share variance (r = 0.48) in this PwMS sample that is not captured solely by Depressed Affect. Further inspection of model mis-fit suggested that the two Somatic Symptoms items ‘I felt everything that I did was an effort (CESD7)’ and ‘I could not get going (CESD20)’ also shared variance not entirely captured by the Somatic Symptoms factor (MI = 11.08). Indices for this model (Model 3) are presented in Table 2 and Figure 2 and suggest a good fit against the criteria. No further modifications were made as these either made trivial improvements in fit or did not make sense theoretically.

**Table 2.** Goodness-of-fit statistics for the models tested – for the 20-item scale.

Model	Description	χ ^2^	df	χ ^2^/df	NNFI/TLI	CFI	RMSEA	SRMR
1	As specified in Radloff1	328.04	164	2.00	0.912	0.924	0.065	0.051
2	Model 1 with correlated error terms for items CESD10 and CESD17	271.26	163	1.66	0.942	0.950	0.053	0.049
3	Model 2 with correlated error terms for items CESD7 and CESD20	255.83	162	1.58	0.949	0.957	0.050	0.046
4	Model 3 tested with the confirmation sample data	240.93	162	1.49	0.957	0.963	0.045	0.047

The re-specified model was then assessed in the confirmation sample (Model 4) and the fit indices suggest a satisfactory fit of this modified 4-factor model. Indeed, the fit indices are slightly better in the confirmation sample than the exploratory one. The modification made in the re-specification process did not imply factorially complex items nor that the basic 4-factor CES-D model was substantively incorrect, so we estimated the standard Cronbach’s Alpha reliability coefficients for the implied CES-D subscales in the confirmation sample. These were 0.81 for Positive Affect, 0.87 for Depressed Affect, 0.73 for Somatic Symptoms and 0.79 for Interpersonal Problems.

## Discussion

The four-factor structure of the CES-D for the 19-item scale was supported by the factor analyses reported here. On the basis of these results, the English version of the CES-D has factorial validity in PwMS. Despite the potential contribution of neuropathology to symptoms, it does appear to have a coherent structure with which to examine depression in PwMS. Consistent with Shafer ^20^, it supports the potential for considering the four factors in research such as that which might consider whether these factors vary as part of the natural course of co-existent depression or indeed that considers whether treatments might differentially affect the factors.

The main limitation of the current study is the typographical error we found in the questionnaire. We did not initially spot the typographical error (item 10 reads “I feel tearful” when it should read “I feel fearful”), and only noticed it after we conducted our initial analyses. It should be noted the compendium of instruments from which the CES-D was obtained is commonly available: it is held in many university and clinical departments. On this account we have contacted the First author of the compendium so notifications might ensue.

Interestingly, after a brief search for studies that give details of the CES-D items used we note that other studies ^36– 39^ have also included this (or a similar) version of the questionnaire, meaning their results will need to be re-evaluated. In fact, one Rasch analysis study using the CES-D in a rheumatoid arthritis sample ^38^ found differential item functioning on this item (which the authors present as “I felt tearful”) for age and gender. The authors do suggest that this finding be replicated before a decision is made to remove this (and one other) item from the scale.

We could not complete a full review of the literature to determine how many other studies have used a version of the CES-D that includes the typographical error, and the impact this has on the findings from these studies. One of the problems is that not all authors report where they obtained the scale from, instead only citing the original reference of the scale. We were, however, able to spot the typo by looking for the word ‘tearful’ when item 10 is mentioned in the paper. On account of the error in the questionnaire, our modelling requires replication with all the full version of the CES-D. Nonetheless, despite this our result at this point are sufficient for us to be confident of how this this instrument would likely perform, that is, consistent with the four-factor solution.

## Data availability

### Underlying data

Consent was not obtained from participants for the sharing of their data, meaning that access to the data is restricted. Those wishing to access the data can apply for access. The data custodian is Prof Chris Fife-Schaw, University of Surrey ( c.fife-schaw@surrey.ac.uk). Access will be provided to researchers at accredited institutions.

## Footnotes

^a^We used the photocopy-permitted master copy of the scale from the Measures in Health Psychology: A User's Portfolio (Johnston, Wright, & Weinman, 1995). An erratum for this measure has now been issued.
