# Supplementary material for: The factor structure of the Center for Epidemiological Study - Depression Scale in people with multiple sclerosis
Source: F1000Res. 2020 Aug 25;9:1038. [Version 1] doi: 10.12688/f1000research.25129.1 (PMC7851711; doi:10.12688/f1000research.25129.1)
Supplement: Peer review history

# Peer review: Reviewer response for version 1

**DOI:** 10.5256/f1000research.27732.r76797

**Published:** 2021-02-01

**Authors:** Bilge Piri Cinar

**Affiliations:** 1 Zonguldak Bülent Ecevit University, Zonguldak, Turkey

1. This depression scale is not used very often in MS. For this reason, it is recommended that the reason for choosing this scale should be briefly explained in the introduction part of the comparison studies with other scales.
2. When the models in the article are examined in the source where values such as SRMR and RMSEA are referred, the models seem sufficient. The general outline of the study is well-designed and the statistical approach has its strengths in general.
3. It is recommended to indicate whether the sample size has been calculated or not.
4. According to a general rule, it is stated that the number of items recommended for the study group to use the factor analysis technique or the sample size five times the number of observed variables (Child, 2006 ^1^). According to Kline (1994 ^2^), although it is recommended to keep the item (variable) ratio 10: 1 for the sample size, it is stated that this ratio can be reduced, but should be at least 2: 1. It is recommended to give information about the sample size in the method section.
5. The suitability of the dataset for factor analysis is recommended to be detailed in the statistics section (normal distribution ...).
6. It would be appropriate to write explanations and abbreviations under the tables and figures.

Is the work clearly and accurately presented and does it cite the current literature?

Yes

If applicable, is the statistical analysis and its interpretation appropriate?

I cannot comment. A qualified statistician is required.

Are all the source data underlying the results available to ensure full reproducibility?

Partly

Is the study design appropriate and is the work technically sound?

Yes

Are the conclusions drawn adequately supported by the results?

Yes

Are sufficient details of methods and analysis provided to allow replication by others?

Partly

Reviewer Expertise:

I am an expert in the field of MS. In all my studies, I definitely consider the presence of depression in patients. In our MS clinic, we routinely use different depression scales in practical life.

I confirm that I have read this submission and believe that I have an appropriate level of expertise to confirm that it is of an acceptable scientific standard.

## References

[1] : The Essentials of Factor Analysis. *Continuum*.2006;3rd Edition: Reference source

[2] : An Easy Guide to Factor Analysis.2014; 10.4324/9781315788135 10.4324/9781315788135

# Peer review: Reviewer response for version 1

**DOI:** 10.5256/f1000research.27732.r71819

**Published:** 2020-10-07

**Authors:** Daphne Kaklamanou

**Affiliations:** 1 Department of Psychology, University of Portsmouth, Portsmouth, UK

This is an interesting article that is looking at the factor structure of the CES-D scale for PwMS. The study has a really good sample size to complete the analysis and draw conclusions from.

 Overall the introduction seems to cover most of the literature  in the area, although the systematic review (Hind *et al.*, 2016 ^1^) on the reliability and validity of depression inventories for PwMS is missing, so the authors might want to check that they have not missed anything. Also it would be good to explain why the authors chose the CES-D scale rather than other depression inventories.

 For the Method section, there seems to be some detail, but more detail of the CES-D scale factor structure and the items would be good and would help understand the discussion in terms of the factors. In terms of the participants, it is clearly stated that this is a study that has taken place over a long period of time but providing a bit more detail (1) on the exact time/year; (2) over how long did they collect the data that are analysed in the study; (3) were there any other measurements collected e.g.  other depression measures or the Gold standard the interview. 

 At some in the method section the authors state that "the CES-D data collected at phase 1 are reported here", I presume they mean the current study.

Is the work clearly and accurately presented and does it cite the current literature?

Partly

If applicable, is the statistical analysis and its interpretation appropriate?

Yes

Are all the source data underlying the results available to ensure full reproducibility?

Partly

Is the study design appropriate and is the work technically sound?

Yes

Are the conclusions drawn adequately supported by the results?

Yes

Are sufficient details of methods and analysis provided to allow replication by others?

Partly

Reviewer Expertise:

I have done work looking at the reliability and validity of depression inventories for PwMS and looked into user experience of depression inventories in PwMS.

I confirm that I have read this submission and believe that I have an appropriate level of expertise to confirm that it is of an acceptable scientific standard.

## References

[1] : The assessment of depression in people with multiple sclerosis: a systematic review of psychometric validation studies. *BMC Psychiatry*.2016;16(1) : 10.1186/s12888-016-0931-5 10.1186/s12888-016-0931-5 27491674PMC4973535
